# Changes in Coronary Physiology by μFR Measurement After DREAMS-3G-Scaffold Implantation

**DOI:** 10.1016/j.jacadv.2025.101944

**Published:** 2025-07-04

**Authors:** Hector M. Garcia-Garcia, Ryan L. Wallace, Adrian Wlodarczak, René J. van der Schaaf, Jan Torzewski, Bert Ferdinande, Javier Escaned, Juan F. Iglesias, Michael Haude, Ron Waksman

**Affiliations:** a Interventional Cardiology, MedStar Washington Hospital Center, Washington, District of Columbia, USA; b Department of Cardiology, Miedziowe Centrum Zdrowia SA, Lubin, Poland; c Department of Interventional Cardiology, OLVG, Amsterdam, The Netherlands; d Faculty of Medicine, Wroclaw University of Science and Technology, Wroclaw, Poland; e Division of Cardiology, Cardiovascular Center Oberallgäu-Kempten, Kempten, Germany; f Department of Cardiology, Ziekenhuis Oost Limburg (ZOL), Genk, Belgium; g Division of Cardiology, Hospital Clinico San Carlos IDISSC, Complutense University of Madrid, Madrid, Spain; h Cardiology Division, University Hospital of Geneva, Geneva, Switzerland; i Medical Clinic I, Rheinland Klinikum Neuss GmbH, Lukaskrankenhaus, Neuss, Germany

**Keywords:** bioresorbable stents, coronary physiology, DREAMS 3G scaffold, late lumen loss, μFR

**Figure:**
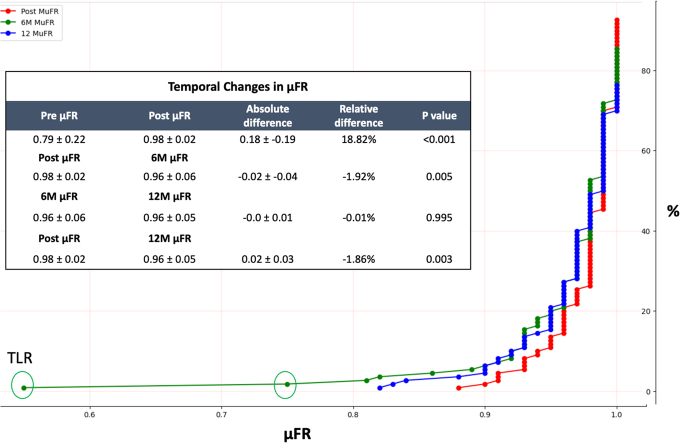

Bioresorbable stent scaffolds provide a potential alternative approach that avoids the long-term presence of a rigid cage that may prevent restoration of coronary artery function and physiology. The DREAMS 3G resorbable magnesium scaffold safety outcomes have been previously demonstrated in the BIOMAG (BIOTRONIK – Safety and Clinical Performance of the Sirolimus-Eluting Resorbable Coronary Magnesium Scaffold System [DREAMS 3G] in the Treatment of Subjects with de Novo Lesions in Native Coronary Arteries)-1 trial.^1^ The third-generation DREAMS 3G scaffold system is composed of resorbable magnesium alloy that resorbs within 12 months and most notably has increased radial strength and thinner struts.^2^ Given this dynamic process, the affected vessel undergoes significant changes in vessel support and flexibility over a 12-month period of resorption. While previous studies have focused on improvements in in-scaffold late lumen loss (LLL) between the DREAMS 3G and previous generation scaffold system, this dynamic process may be better reflected by nonanatomical outcomes.^2^

**What is the clinical question being addressed?**

Is angiography-derived physiology changes feasible and clinically meaningful?

**What is the main finding?**

μFR measurements significantly improved to clinically normal levels after DREAMS 3G scaffold implantation, and these levels of μFR improvement are sustained to 12-month follow-up.

We performed a post hoc analysis of the BIOMAG-1 study to investigate the temporal changes in noninvasive Murray law-based quantitative flow ratio—μFR (Pulse Medical Technology Inc) measurements after DREAMS 3G implantation and compare them to in-scaffold LLL over the same time frame. Local ethics committees at participants’ sites approved the study protocol.

In the BIOMAG-1 trial, a total of 116 patients were enrolled. Full inclusion and exclusion criteria can be found in the original BIOMAG-1 trial.^1^ μFR is a noninvasive Murray law-based quantitative flow ratio using one view in this study. Patients underwent μFR assessment before and immediately after DREAMS 3G implantation during index procedure. μFR was also measured at follow-up 6- and 12-month angiographic reassessment. A hemodynamically significant μFR of clinical importance is ≤0.8. An independent core laboratory (MedStar Cardiovascular Research Network) performed the analysis. In-scaffold LLL was measured by quantitative coronary angiography and was assessed at 6 months and 12 months.

After normality testing was applied, the μFR measurements between different time points were compared using a 2-tailed *t*-test. The level of significance (alpha) was set at 0.05 for all statistical tests. Absolute difference was calculated as the mean difference between μFR measurements at various time intervals. Relative difference was also obtained by calculating (1 − [μFR at time point 1/μFR at time point 2]) × 100, representing a percent change between the time intervals. Coefficient correlations between μFR and LLL were calculated. All statistical analyses were performed using Jupyter Lab version 3.4.4 based on Python version 3.9.13 and SAS (IBM) 9.4.

The full baseline demographics of the patient population and lesion characteristics can be found in the original manuscript.^1^ After implantation of DREAMS 3G, μFR increased from a clinically abnormal mean measurement of 0.79 ± 0.22 to a clinically normal mean measurement of 0.98 ± 0.02 (*P* < 0.001). Compared to baseline μFR, there was a decrease in μFR at 6 months (0.98 ± 0.02 vs 0.96 ± 0.06; *P* = 0.005), but it remained above the clinically abnormal μFR threshold of ≤0.8. The μFR measurements compared between 6 and 12 months showed no significant difference (0.96 ± 0.06 vs 0.96 ± 0.05; *P* = 0.995). The μFR immediately post DREAMS 3G implantation compared to 12-month follow-up was significantly reduced (0.98 ± 0.02 vs 0.96 ± 0.05; *P* = 0.003) but remained well above the clinically abnormal μFR threshold of ≤0.8. Relative difference in μFR immediately post DREAMS 3G implantation compared to 12 months showed a 1.86% decrease in μFR, after an initial increase in μFR of 18.82% immediately postimplantation. See Figure 1 for more details regarding temporal changes in μFR. Of the patients included in the analysis, all but 3 patients maintained a μFR above 0.80 over 12-month follow-up (Figure 1). These patients had a target lesion revascularization procedure. μFR measurement prior to bioresorbable scaffold did not correlate with LLL at either 6-month (r = −0.040) or 12-month follow-up (r = 0.08). Furthermore, μFR measurement had a negative correlation when comparing both 6-month μFR with 6-month LLL (r = −0.48), as well as 12-month μFR with 12-month LLL (−0.53).

**Figure 1:**
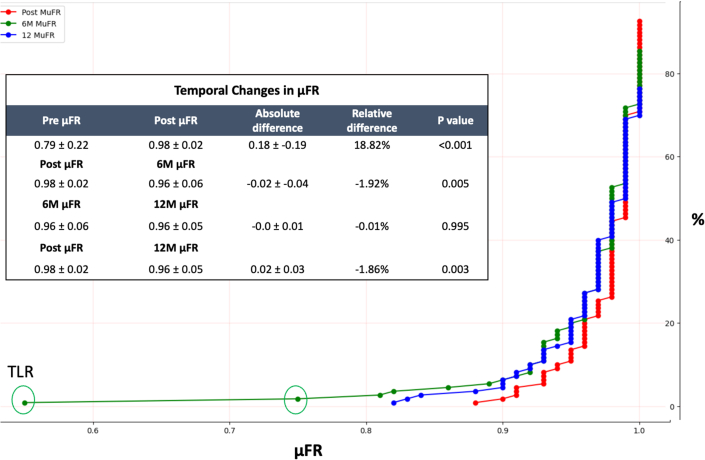
Cumulative Curves for μFR Postintervention, 6 Months and 12 Months Table displays the mean ± SD of the Pre: μFR preintervention; Post: μFR postintervention; 6Mo: μFR at 6Mo FU; 12Mo: μFR at 12Mo FU; absolute difference was calculated as mean difference between the 2 time points; relative difference was calculated as (1 − [μFR at time point 1/μFR at time point 2]) × 100.

Previous anatomical assessment of this same cohort has shown that while there is lower scaffold LLL compared to the previous bioresorbable scaffold generation, there remains a scaffold LLL of 0.24. ± 0.36 mm (95% CI: 0.02-0.19) over a 12-month follow-up period.^1^ However, LLL may not always predict clinical outcomes,^3^ and previous studies have shown strong correlation between initial improvement in post-percutaneous coronary intervention fractional flow reserve and patient quality of life improvements and angina.^4^ In our study that included stable patients with strict inclusion and exclusion criteria and had excluded large bifurcations, heavy calcifications, and acute myocardial infarction, μFR measurement was significantly improved from preimplantation to immediately postimplantation, and although there was a statistically significant decrease in μFR at 12-month follow-up, all μFR measurements remained well above the clinically accepted abnormal threshold of μFR ≤0.80. Furthermore, there was a moderate negative correlation between the μFR and late luminal loss at 6 and 12 months. In addition to correlation with symptom relief, coronary physiology may better reflect overall vessel health compared to anatomical outcomes.

In conclusion, our findings suggest that μFR is significantly improved to clinically normal levels after DREAMS 3G scaffold implantation and that these levels of μFR improvement are sustained to 12-month follow-up.

## Funding support and author disclosures

The study was funded by 10.13039/501100005035Biotronik AG. Drs Garcia-Garcia, Wallace, were core laboratory members, the remaining authors were investigators of the trial. Dr Haude has received grants/contracts from 10.13039/501100005035Biotronik, Cardiac Dimensions, Orbus Neich, and Philips; has received consulting fees from 10.13039/501100005035Biotronik, Cardiac Dimensions, Shockwave Medical, and Orbus Neich; has received honoraria/speaker fees from 10.13039/501100005035Biotronik, Cardiac Dimensions, Shockwave Medical, Orbus Neich, and Philips; support to attend meetings/travel support from 10.13039/501100005035Biotronik; is a steering committee member of the BIOSOLVE and BIOMAG trials; and is a past president of EAPCI. Dr Torzewski has received grants and contracts from 10.13039/100000046Abbott paid to his institution; has received speaker honoraria and support for attending meetings from 10.13039/501100005035Biotronik; and is an associated editor of Cardiovascular Biologics and Regenerative Medicine and Frontiers in Cardiovascular Medicine. Dr Escaned has received personal fees/speaker honoraria from 10.13039/100000046Abbott, 10.13039/100008497Boston Scientific, Philips, and Shockwave, patents from Shared; and participated in advisory boards of 10.13039/100000046Abbott and Philips. Dr Iglesias has received grants or contracts to his institution from Terumo Corp, Concept Medical, Biotronik, SMT, Abbott Vascular, and Philips Volcano; has received consulting fees from Biotronik, Medtronic, Cordis, and ReCor Medical; and has received speaker fees/honoraria from Terumo Corp, Biosensors, Biotronik, Concept Medical, Bristol Myers Squibb/Pfizer, Novartis, Cordis, AstraZeneca, Medtronic, Penumbra, ReCor Medical and Philips Volcano; and support to attend meetings from Biotronik, and Medtronic. Dr Garcia-Garcia has received grants or contracts from 10.13039/100004374Medtronic, 10.13039/501100005035Biotronik, 10.13039/100000046Abbott, Neovasc, Corflow, Philips, Chiesi (paid to institution); has received consulting fees from 10.13039/100008497Boston Scientific and ACIST; and participated in DSMB/advisory board of the VIVID study. Dr Waksman has received grants or contracts from Amgen, 10.13039/501100005035Biotronik, 10.13039/100008497Boston Scientific, 10.13039/100004374Medtronic, and Philips IGT; has received consulting fees from 10.13039/100011949Abbott Vascular, 10.13039/501100005035Biotronik, 10.13039/100008497Boston Scientific, Cordis, 10.13039/100004374Medtronic, Philips IGT, Pi-Cardia Ltd, Swiss Interventional Systems/SIS Medical AG, Transmural Systems Inc, and Venous MedTech; has received honoraria from AstraZeneca; participated in DSMB/advisory boards of 10.13039/100011949Abbott Vascular, 10.13039/100008497Boston Scientific, 10.13039/100004374Medtronic, Philips IGT, and Pi-Cardia Ltd; and is an investor from MedAlliance and Transmural Systems Inc. All other authors have reported that they have no relationships relevant to the contents of this paper to disclose.
